# An Atlas of Thyroid Hormone Responsive Genes in Adult Mouse Hypothalamus

**DOI:** 10.1210/endocr/bqaf084

**Published:** 2025-04-30

**Authors:** Shijia Wu, Julien Dellinger, Suzy Markossian, Yves Dusabyinema, Romain Guyot, Sandrine Hughes, Denise Aubert, Marie Fackeure, Karine Gauthier, Benjamin Gillet, Wenzheng Jiang, Frédéric Flamant

**Affiliations:** Institut de Génomique Fonctionnelle de Lyon, UMR5242, École Normale Supérieure de Lyon, INRAE USC1370, CNRS, Lyon 69007, France; Shanghai Key Laboratory of Regulatory Biology, Institute of Biomedical Sciences and School of Life Sciences, East China Normal University, Dongchuan Road 500, Shanghai 200241, China; Institut de Génomique Fonctionnelle de Lyon, UMR5242, École Normale Supérieure de Lyon, INRAE USC1370, CNRS, Lyon 69007, France; Institut de Génomique Fonctionnelle de Lyon, UMR5242, École Normale Supérieure de Lyon, INRAE USC1370, CNRS, Lyon 69007, France; Institut de Génomique Fonctionnelle de Lyon, UMR5242, École Normale Supérieure de Lyon, INRAE USC1370, CNRS, Lyon 69007, France; Institut de Génomique Fonctionnelle de Lyon, UMR5242, École Normale Supérieure de Lyon, INRAE USC1370, CNRS, Lyon 69007, France; Institut de Génomique Fonctionnelle de Lyon, UMR5242, École Normale Supérieure de Lyon, INRAE USC1370, CNRS, Lyon 69007, France; Institut de Génomique Fonctionnelle de Lyon, UMR5242, École Normale Supérieure de Lyon, INRAE USC1370, CNRS, Lyon 69007, France; Institut de Génomique Fonctionnelle de Lyon, UMR5242, École Normale Supérieure de Lyon, INRAE USC1370, CNRS, Lyon 69007, France; Institut de Génomique Fonctionnelle de Lyon, UMR5242, École Normale Supérieure de Lyon, INRAE USC1370, CNRS, Lyon 69007, France; Institut de Génomique Fonctionnelle de Lyon, UMR5242, École Normale Supérieure de Lyon, INRAE USC1370, CNRS, Lyon 69007, France; Shanghai Key Laboratory of Regulatory Biology, Institute of Biomedical Sciences and School of Life Sciences, East China Normal University, Dongchuan Road 500, Shanghai 200241, China; Institut de Génomique Fonctionnelle de Lyon, UMR5242, École Normale Supérieure de Lyon, INRAE USC1370, CNRS, Lyon 69007, France

**Keywords:** single nuclei analysis, thyroid hormone, hypothalamus, bulk RNA-seq, single nuclei RNA-seq

## Abstract

We present an atlas of genes that respond to thyroid hormone in the adult mouse mediobasal hypothalamus. Based on droplet-based single nuclei RNA-seq method and batch transcriptome analyses, the atlas lists putative target genes of the hormone nuclear receptors in 20 different types of neuronal and glial cells. The transcriptional regulation of these genes varies extensively across neuronal and glial cell types. However, while astrocytes appear to be highly sensitive to thyroid hormone stimulation, differentiated oligodendrocytes are relatively insensitive. This atlas is expected to promote future investigations into the molecular and cellular mechanisms that underlie the numerous functions of thyroid hormone in the hypothalamic circuits.

The hypothalamus represents a major interface between the brain and the periphery. It integrates external biochemical and sensory input to maintain homeostasis under various circumstances, controlling pituitary secretions, blood pressure, energy metabolism, sleep, appetite and reproduction. It is also the location of the central circadian and seasonal clocks. Its extreme neuroanatomical complexity, interconnecting 15 nuclei, enables a number of cross-talks between these functions (1).

The thyroid hormones (TH, including thyroxine, or T4, and its more active metabolite 3,3′,5-triiodo-L-thyronine or T3) exert a broad influence on hypothalamic functions. Several regulations have been documented, suggesting that the hypothalamus is one of the main targets of T3 action: (i) T3 downregulates thyrotropin-releasing hormone (TRH) secretion, which stimulates in turn the secretion of TSH by thyrotroph cells in the pituitary, and thus exerts a negative feedback regulation on the hypothalamic-pituitary-thyroid axis (2); (ii) T3 exerts local control on metabolism and energy balance by stimulating the sympathetic nervous system (3); (iii) T3 maintains the set point of body temperature (4); (iv) T3 inhibits food intake, refraining the activity of proopiomelanocortin (POMC) neurons (5) while activating neuropeptide Y (NPY) neurons (6); (v) T3 is a major component of the seasonal clock, coupling the length of the day with seasonal behavior (7); and (vi) T3 is also a relay to inflammatory response in pathological situations (8, 9).

The T3 content of the mediobasal hypothalamus is not directly coupled to the circulating level of TH. It can be enhanced by the conversion of circulating T4 by type 2 deiodinase, an enzyme that is present mainly in the tanycytes lining the wall of the lower part of the third ventricle. On the contrary, when T3 is in excess, the catabolic enzyme type 3 deiodinase is expressed in other cell types, including neurons and tanycytes located in the upper part of the ventricle, and the local level of T3 decreases. This metabolism allows rapid changes in T3 local content, while serum concentration remains stable. This also generates a local heterogeneous distribution of T3, which probably has a paracrine influence on specific neuronal cell populations in specific nuclei (10).

T3 exerts its action by binding to nuclear receptors, TRα1 and TRβ1/2 which are ligand-dependent transcription factors, collectively called TRs (11). They are encoded by the *Thra* and *Thrb* genes, which are expressed in many cell types, including virtually all hypothalamic cell types. Therefore, a complete understanding of T3 function in the hypothalamus represents a daunting task. The full characterization of TR target genes in each hypothalamic cell types would represent a major achievement and a significant progress in the understanding of many physiological functions. As a first step of this program, we establish here an atlas of T3-responsive genes in the mediobasal hypothalamus of adult mice at cellular resolution. This in-depth analysis identifies the most responsive cell types and lists putative TR target genes in each of them.

## Methods

### Animals

The research project was approved by a local ethics committee for animal experimentation (C2EA015) and subsequently authorized by the French Ministry of Research (Project #39805-2022110119433980 v4). Two groups of C57Bl6/j mice were fed for 2 weeks with iodine-deficient food supplemented with 0.15% of propyl-thiouracyl (Envigo ref TD.95125) to cause deep hypothyroidism. The first group of mice, referenced TH^high^, received a single intraperitoneal TH injection (40 μg T4 + 4 μg T3 dissolved in 200 µL of phosphate buffer saline). The second group of mice, referenced TH^low^, was injected with 200 µL of phosphate buffer saline as control. Mediobasal hypothalami were dissected the next day, flash frozen using liquid nitrogen and stored at −70 °C.

### Bulk Whole Cell RNA-seq From Sorted Hypothalamus Astrocytes

Hypothalami were dissected from 2 groups of 5 wild-type mice prepared as above. Astrocytes were isolated by magnetic sorting using the Anti-GLAST (ACSA-1) MicroBead Kit (Miltenyi Biotec Cat# 130-123-555, https://www.antibodyregistry.org/AB_2811532). The cDNAs were directly prepared from individual whole cell lysates using the whole cell RNA SMART-Seq V4 Ultra Low Input Kit (Takara). Sequencing libraries were then prepared from 1 ng of cDNA using the Nextera XT DNA Library Preparation Kit (Illumina) with Unique Dual Indexes. Quantification and qualification of dual indexed libraries were performed with both Qubit 4.0 (HS DNA Kit, Thermo Fisher) and Tapestation 4150 equipment using the D5000 ScreenTape System (Agilent). Qualified libraries were pooled in an equimolar manner and then sequenced on a NextSeq 500 platform (Illumina) according to the manufacturer's instructions. Sequencing was performed with High Output reagents in single-end and dual indexing, for a total of 72, 10 and 10 cycles run for Read 1, i7 and i5 index respectively. Raw sequencing data were deposited at EMBL-EBI, accession no. E-MTAB-15049.

### Bulk Nuclei RNA-seq From Sorted GABAergic Neurons

Transgenic *Gad2Crex ROSA-GSL10gfp^lox^* mice expressing a green fluorescent protein (GFP) in the nuclei of GABAergic neurons were treated as above. In these mice, the *Gad2Cre* driver (IMSR Cat# JAX:010802, RRID: IMSR_JAX:010802) drives Cre/loxP recombination in GABAergic neurons. It is combined with *t*he *ROSA-GSL10gfp^lox^* reporter transgene (12) for the conditional expression of a fusion protein based on the ribosomal L10a protein (13). Hypothalami were frozen individually in liquid nitrogen immediately after dissection. Samples were thawed and resuspended in 1.5 mL of lysis buffer (Nuclei EZ Lysis Buffer, Sigma Aldrich) and transferred in a 2-mL Kimble Dounce tissue grinder (Sigma Aldrich) for cell lysis (25 strokes with A pestle and 25 strokes with B pestle, on ice). Suspensions were transferred into tubes containing 2.5 mL of cold lysis buffer, incubated 5 minutes on ice and centrifuged (5 minutes 500*g* 4 °C, swinging rotor). Supernatants were discarded and nuclei pellets were washed once with 4 mL Nuclei EZ Lysis Buffer and once with 4 mL of Nuclei Suspension Buffer (NSB: phosphate buffer saline without Mg^2+^ and Ca^2+^; 1% nuclease free BSA (Sigma); 200 U/mL RNAsin (Promega). Nuclei pellets were resuspended in 0.3 mL NSB and filtrated on a 35 μm cell strainer (Corning). Then 1 μg of DAPI (Sigma Aldrich) was added to nuclei suspensions for DNA staining, allowing FACS sorting of singlets and not of aggregates of several nuclei. All nuclei resuspensions were achieved by pipetting 10 times with 1 mL of buffer. All GFP positive single nuclei were sorted (FACS Aria IIµ cytometer; BD Biosciences) in low-binding Eppendorf tubes containing 0.5 mL NSB. Nuclei were pelleted (5 minutes 2.10^4^ g). Supernatant were discarded and nuclei resuspended in 350 μL RNA lysis buffer containing β-mercaptoethanol. RNA was extracted (RNeasy Micro Kit, Qiagen) and quantified using Tapestation 4150 (Agilent). The cDNAs were prepared from 1 ng of RNA using the RNA SMART-Seq V4 Ultra Low Input Kit (Takara Bio). Libraries were constructed and sequenced as described above. More than 24 M reads by sample were obtained. Raw sequencing data were deposited at EMBL-EBI, accession no. E-MTAB-15050.

### Bulk RNA-seq Data Analysis

The 2 bulk RNA-seq datasets were analyzed using the European Galaxy platform (14) (https://usegalaxy.eu/ is maintained by the Freiburg Galaxy Team). Raw reads were mapped on the mouse genome (GRCm38 mm10 assembly), using Bowtie2 (Galaxy Version 2.5.3 + galaxy1) (15). Htseq-count (Galaxy Version 2.0.5 + galaxy0) (16) was then used to assign reads to genes (feature type: genes for GABAergic neurons nuclei, or exons for astrocytes). Differential gene expression analysis was performed with Deseq2 (Galaxy Version 2.11.40.8 + galaxy0) (17). Unsupervised principal component analysis, performed on gene count table of all samples from each project, identified one outlier library for astrocyte RNA-seq, one of the TH^low^ samples, which was excluded from further analysis. Hierarchical clustering was performed using Euclidian distance and Ward's method with Clustvis (18).

### Nuclei Isolation From Hypothalamus, Single Nuclei RNA Library Preparation, and Sequencing

Three hypothalamus samples (2 males, 1 female) were pooled from each group of mice, and nuclei were extracted as above except that the final centrifugation step was modified (5 minutes 300 g 4 °C swinging rotor). This step was optimized to minimize nuclei aggregation, which might produce clustering artifacts. For each condition, 2.10^5^ nuclei were sorted and centrifuged at low speed to prevent nuclei aggregation. Supernatant volume was reduced to 100 μL before nuclei resuspension. Concentrations (1300 nuclei/μL) were determined with a Malassez hemometer. Quality control was later performed with ImageStream X cytometer (ISX, Luminex) to define the distribution of nuclei area (52 ± 2 μm^2^), diameter (0.8 ± 0.02 μm) and circularity (score 10.8 ± 0.5). Immediately after concentration determination, around 2.10^4^ nuclei were partitioned to produce gel beads-in-emulsion (GEMs) using the Chromium Controller (10 × Genomics). Then, reverse transcription, cDNA recovery, cDNA amplification, and library construction were performed using the Chromium Next GEM Single Cell 3′ Reagent Kits v3.1 (10 × Genomics) following the manufacturer's protocols. Quantification and qualification of dual indexed libraries, referenced Lib^THlow^ and Lib^THhigh^, were done with both Qubit 4.0 (High Sensitivity DNA Kit, Thermo Fisher) and Tapestation 4150 assays (D5000 ScreenTape reagents, Agilent). Qualified libraries were pooled in an equimolar manner and then sequenced on the NextSeq 500 platform (Illumina) using High Output reagents, according to the manufacturer's instructions (Illumina), with paired-end sequencing (28 and 120 cycles for reads R1 and R2, respectively) and dual indexing (10 cycles for each index).

### Single Nuclei RNAseq Data Analysis

The Illumina raw sequencing data were analyzed with the dedicated Genomics set of pipelines, Cell Ranger (v7.1.0). Bcl files were first demultiplexed and converted to fastq files with the “mkfastq” pipeline. Single-cell feature-barcode matrices were then generated from fastq files with the count pipeline using the mouse genome (GRCm38 release 102) retrieved from Ensembl. Because single nuclei sequencing data are enriched in intronic reads, the include-introns argument was added to count reads falling in the intronic regions. More than 10^4^ nuclei were identified by Cell Ranger in each library. It should be noted that over 30% of raw reads were mapped in antisense to genes and were therefore not included in the analyses. However, this high proportion is expected for Chromium Single Cell 3' Gene Expression v3.1 libraries when applied to suspensions of mouse brain nuclei (see Technical Note 10 × CG000376), with multiple mechanisms leading to these artifacts.

For each library, the filtered-feature_bc_matrix data (barcodes, features and matrix) were analyzed further with the Seurat toolkit v5 (19) considering a min.cells parameter equal to 3. Various filters were applied to reduce noise (nfeature_RNA parameter ranging from 200 to 5 000 for Lib^THlow^, 200 to 4 000 for Lib^THhigh^), and exclude doublets (DoubletFinder) (20) and cells with high mitochondrial content (>5% of reads). After these cleaning steps, 9504 nuclei and 9154 nuclei were retained for Lib^THlow^ and Lib^THhigh^, respectively.

The 2 libraries were merged and normalized using the SCT normalization method, then integrated with the harmony method (21) in order to facilitate accurate comparative analysis across both datasets. The 25 first principal components were used to calculate a neighbor graph (FindNeighbors), based on which Louvain clustering was performed with a resolution of 0.3 identifying 21 biologically relevant cell clusters (FindClusters). The clusters annotation was performed manually, taking advantage of the HypoMap atlas (22).

The top 25 principal components were embedded onto 2 dimensions based on the UMAP algorithm using the RunUMAP function with 20 neighbors. The differentially expressed features were identified between both condition for each cluster (FindMarkers).

### Quantitative Reverse Transcriptase Polymerase Chain Reaction

Total RNA was converted to cDNA using MMLV reverse transcriptase (Promega, Wisconsin, USA). Quantitative reverse transcriptase polymerase chain reaction (RT-qPCR) was performed using SYBRGreen mix (BioRad iQ supermix). The results were analyzed according to the ΔΔCT method (23). The housekeeping gene Hprt was used as the reference.

## Results

### Single Nuclei RNA-seq

We prepared two 10 × Genomics droplet-based libraries for single nuclei RNA (snRNA)-seq, Lib^THlow^ and Lib^THhigh^, to address the response of mediobasal hypothalamus cells to TH over 24 hours (see “Methods”). Previous experiments indicate that this setting maximizes the chance to detect a robust hormonal response, while the relatively short duration of TH treatment minimizes its indirect effects, secondary to its systemic influence.

Deep sequencing (310 × 10^6^ and 260 × 10^6^ reads respectively for Lib^THlow^ and Lib^THhigh^) provided sufficient data after filtering to explore the transcriptome of 18 658 cells (9 504 and 9154 cells; 28 717 and 27 868 total unique genes, 2250 and 1872 median genes per cell for Lib^THlow^ and Lib^THhigh^, respectively). In particular, low mitochondrial level (<1%) and doublets were observed in datasets confirming the good quality of sample preparation.

### Cell Clusters Identification

**Figure 1.**
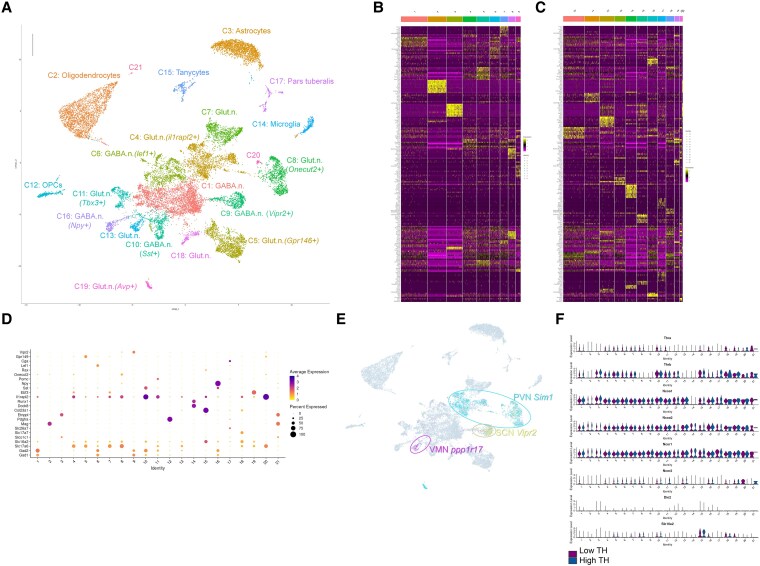
Single nuclei RNA-seq analysis of the TH response of hypothalamus from hypothyroid mice. A, UMAP representing the data collected from 18 658 nuclei, embedding Lib^THlow^ and Lib^THhigh^ and colored by cluster. Clusters are numbered by decreasing nuclei numbers and associated with main markers, as defined by differential expression analysis (see Table 1 and Supplementary Fig. S2) (24). B, Heatmap of expression for genes differentially expressed between clusters, for clusters 1-9. High expression in yellow, low expression in pink. C, Heatmap for clusters 10-21. D, Dot plot for a selection of genes marking each cluster. Genes were selected on the heatmap and to favor comparison with previously published data. E, Coincidence of neuronal population with specific hypothalamic nuclei. *Sim1* is only expressed in neurons of the paraventricular nucleus (PVN) and is found in cluster 4, 8, and 19. The neurons in cluster 9 that express *Vipr2* mainly belong to the suprachiasmatic nucleus (SCN) while *Ppp1r17*-expressing neurons mainly belong to the ventromedial nucleus (VMN). F, Violin plot summarizing the distribution in the different clusters of cells expressing the main genes of the TH signaling pathway. *Thra* and *Thrb,* respectively encode TRα1, TRβ1/2, the nuclear receptors of T3. *Ncoa1* and *Ncoa2* encode histone acetyltransferases that act as TR coactivators*. Ncor1* and *Ncor2* encode the histone-deacetylase corepressors which interact with the unliganded TR. *Dio2* encodes type 2 deiodinase, which converts T4 to T3. *Slc16a2* encodes the Mct8 transporter, which favors crossing of the brain-blood barrier by TH. Note that only a small fraction of oligodendrocytes (cluster 2) expresses the *Thra, Thrb, Ncoa1,* and *Ncoa2* genes at detectable levels.

We used the Seurat package to cluster the integrated datasets and to visualize two-dimensional uniform manifold approximations and projections (UMAPs). Clustering can be operated with arbitrary resolution, as the definition of cell types relies on hierarchical classification. However, high resolution reduces the number of cells per cluster and reduces the statistical power of differential gene expression analysis. A preliminary analysis indicated that setting the clustering resolution to 0.3 maximizes the chance to discover differentially expressed genes (DEGs) in each cell cluster. At this resolution, 21 cell clusters were defined and ranked according to the number of cells that they contain (Fig. 1A). Examples of expression patterns allowing for cluster discrimination are given in Supplementary Fig. S1 (24). The contribution of Lib^THlow^ and Lib^THhigh^ was balanced except for cluster 15, for which more nuclei were found in Lib^THhigh^, and for cluster 18, where the situation was opposite (Table 1). After a global search for markers for each cluster (Fig. 1B and 1C), we used HypoMap (22) and the Allen Brain Cell Atlas (25) as references to select the most relevant markers and identify the cell types corresponding to each cluster (Fig. 1D). Except for cluster 21, which contains a very small number of cells, this allowed for an unambiguous identification of cell types (Table 1). The analysis of genes expressed in specific hypothalamic nuclei indicates that some types of neuronal cells might be present in a single location (Fig. 1E). For example, the *Sim1* gene, which is mainly expressed in the paraventricular nucleus, is detected in clusters 4 and 19. It is also expressed in cluster 8 but the concomitant expression of *Onecut2* and *Myo5b* rather suggests a main contribution of the ventro-medial nucleus. Cluster 9 gathers GABAergic neurons expressing *Vipr2* which are only found in the suprachiasmatic nucleus. Ventromedial hypothalamic nucleus neurons which express the *ppp1r17* gene are in cluster 13. Therefore, the snRNA-seq dataset provides spatial information, and might be used to predict the response of specific hypothalamic nuclei to TH stimulation. To favor this neuroanatomical interpretation of the data, we presented the data as violin plots for marker genes commonly used in histological analyses (Supplementary Fig. S2) (24). We also plotted the expression level of the main genes of the TH signaling pathway across the cell clusters (Fig. 1F). As expected, *Thra* and *Thrb* and the genes encoding their main coactivators and corepressors were broadly expressed, with different patterns. Only one gene encoding a TH transporter, *Slc16a2* encoding MCT8, was expressed at a significant level, and its expression was restricted to a few cell clusters.

**Table 1.** Characteristics of single nuclei RNA-seq clusters

Seurat cluster	Number of cells		Mean gene count	Category*^a^*	Main markers	Main location*^b^*	No. of DEG	Fold-change >0.66	
	Lib^THlow^	Lib^THhigh^						Up	Down
**1**	1842	1632	3019	**GABA.n.**	***Gad2***		225	7	17
**2**	1306	1144	581	**Oligod.**	***Mag***		57	13	8
**3**	1010	1068	1430	**Astrocytes**	***Slc1a3***		167	34	30
**4**	949	886	1654	**Glut.n.**	***Il1rapl2***	PVN	119	3	16
**5**	802	836	2968	**Glut.n.**	***Fezf1***	DMN	126	10	26
**6**	668	658	4087	**GABA.n.**	***Lef1***		39	2	8
**7**	579	442	3909	**Glut.n.**	***Lmx1a***		38	3	12
**8**	408	567	3036	**Glut.n.**	***Onecut2,* *Myo5b***	VMN	23	2	9
**9**	320	302	1868	**GABA.n.**	***Vipr2, Syt10***	SCN	0	0	0
**10**	259	323	3064	**GABA.n.**	***Sst***	ARC	10	0	5
**11**	192	225	3145	**Glut.n.**	***Tbx3, Pomc***	ARC	7	1	4
**12**	221	179	2837	**OPCs**	***Pdgfra***		3	0	2
**13**	154	155	3651	**Glut.n.**	***Tafa4 Ppp1r17***	AHN VMN	0	0	0
**14**	150	154	967	**Microglia**	***Aif1***		1	0	1
**15**	88	197	3569	**Tanycytes**	***Rax, Col23a1***		9	1	8
**16**	121	160	4531	**GABA.n.**	***Npy***	ARC	2	0	1
**17**	107	114	1116	**Pars tub.**	***Tshb***	Pituitary	0	0	0
**18**	210	10	3443	**Glut.n.**	***Slc17a7***		8	8	0
**19**	83	59	2777	**GABA.n.**	***Avp***	PVN	3	2	1
**20**	18	41	3348	**Glut.n.**	***Tac2***	ARC	0	0	0
**21**	17	2	2576	**ND**			0	0	0
**Total**	**9504**	**9154**					**653**	**68**	**134**

The main markers derive from Fig. 1B-1D. Heading abbreviations: DEG, differentially expressed gene; Lib, library.

^*a*^Category abbreviations: GABA.n., GABAergic neurons; Glut.n., glutamatergic neurons; OPCs, oligodendrocytes precursor cells.

^*b*^Main location abbreviations: AHN, anterior hypothalamus nucleus; ARC, arcuate nucleus; DMN, dorsomedial nucleus; PVN, paraventricular nucleus; SCN, suprachiasmatic nucleus; VMN, ventromedial nucleus.

### snRNA-seq Differential Gene Expression Analysis

**Figure 2.**
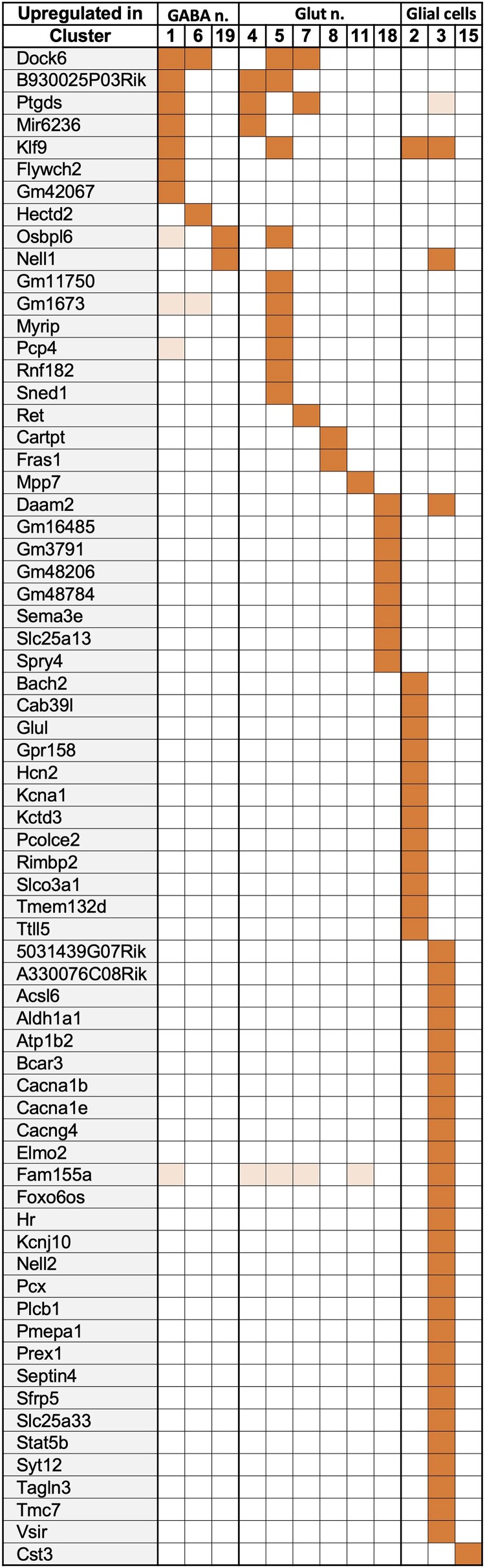
Genes upregulated after TH treatment. Differential expression analysis identified 68 genes for which the log2 fold-change >0.66 and an adjusted *P* value <.05 in at least one cell cluster (dark orange). Light orange corresponds to adjusted *P* value <.05 and a lower log2 fold-change. Note the higher representation of cluster 3 (astrocytes).

**Figure 3.**
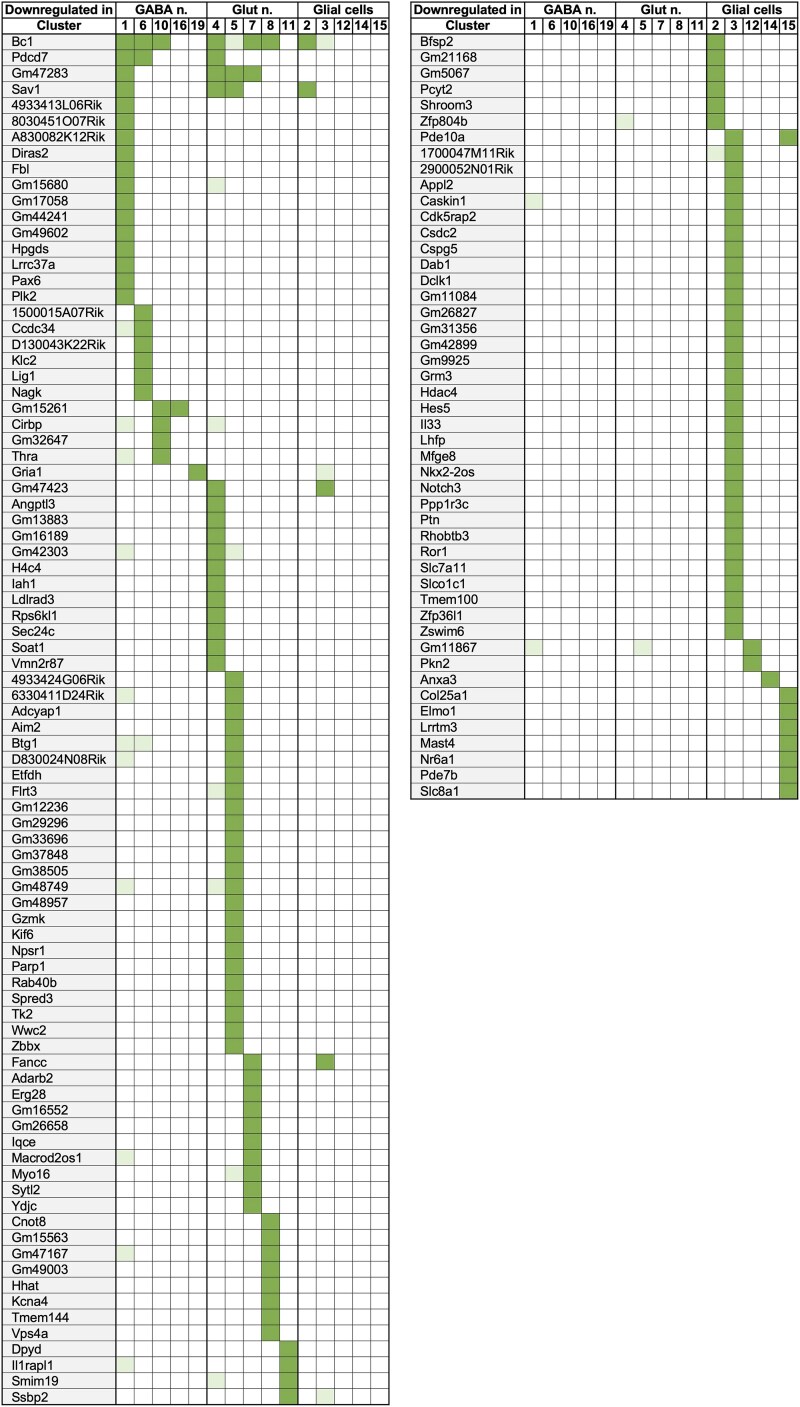
Genes downregulated after TH treatment. Differential expression analysis identified 136 genes for which the log2 fold-change < −0.66 and an adjusted *P* value <.05 in at least one cell cluster (dark green). Light green corresponds to adjusted *P* value <.05 and a lower log2 fold-change.

Table 1 summarizes the result of an analysis that identified a total of 653 differentially expressed genes (adjusted *P* value <.05 DEGs) between Lib^THlow^ and Lib^THhigh^ in the different clusters. Complete results are in Supplementary Table S1 (24) (note that for cluster 18, the unbalance between Lib^THlow^ and Lib^THhigh^ contributions might bias the results). This number drops to 202 if an additional threshold is applied for the fold-change (log2 fold-change >0.66 or < −0.66; Fig. 2 and Fig. 3 for up- and downregulated genes respectively). Few genes were regulated in more than one cluster and a recent compilation indicates that only some of these (*B930025P03Rik, Daam2, Fancc, Klf9, Osbpl6, Ptgds, Pdcd7*) have been repeatedly identified as TH-responsive genes in other tissues or cell types (26). However, a large fraction of these DEGs (82%) was found in only one cell cluster, indicating that different cell types display different responses to TH.

### Subclustering

The chosen resolution produces 7 large cell clusters (>10^3^ cells) which might hide a heterogeneity in TH response. To address this possibility, we performed an additional step of clustering for these 7 clusters, optimizing again to favor the discovery of DEGs. At low resolution (0.1), this subclustering step allowed us to identify 75 additional DEGs, of which 63 were not identified in another cluster (+10%; Supplementary Table S2) (24). Interestingly, 26 of these were found in astrocytes (cluster 3), suggesting that cells within this population differ in their response to TH.

### Oligodendrocytes Are Less Sensitive to TH

The capacity to detect DEGs within a cluster is expected to decrease with the number of cells included in the cluster that is considered, as the statistical power of the differential analysis decreases. To illustrate this trend, we performed subclustering at various resolution to subdivide cluster 3 in cell groups of different size. This showed that a linear relationship exists between the number of cells in a cluster, or subcluster, and the number of DEGs, and that differential expression can no longer be detected when the number of cells in a subcluster is below 200 (Supplementary Fig. S3A) (24). The same relation was observed when all clusters were plotted (Supplementary Fig. S3B) (24). However, some deviations to this trend were evident, indicating that the sensitivity to TH varies across cell types. In particular, oligodendrocytes (cluster 2) stood out as less responsive to TH stimulation, compared to GABAergic neurons (cluster 1) and astrocytes (cluster 3) (Table 1). This low sensitivity of oligodendrocytes was correlated with a reduced expression of *Thra, Thrb, Ncoa1,* and *Ncoa2* encoding the main elements of the TH signaling pathway: the TRα1 and TRβ1/2 nuclear receptors, and their coactivators with histone acetyl transferase activity (Fig. 1F).

### Comparisons With Previous Single Nuclei Analysis.

We compared our snRNA-seq data with 2 datasets, which were recently published for related experimental settings (Supplementary Table S3) (24). The first is a snRNA-seq analysis of the hypothalamus from heterozygous mice carrying the dominant-negative *Thra^R384C^* mutation, which inhibits the response to TH from early developmental stages in many tissues (27). We found very limited overlap between the lists of DEGs resulting from our experiment and this one. Notably, the analysis of mutant mice mainly pinpointed a large set of deregulated genes in oligodendrocytes that we did not find to be TH responsive in hypothyroid mice. Thus, the deregulations found in the oligodendrocytes of mutant mice are not direct consequences of altered TH signaling. They appear only after the prolonged and ubiquitous expression of the TRα1^R384C^ receptor and might correspond to a developmental defect.

The overlap was also limited between our data set and the results of a snRNA-seq analysis performed on the neocortex of euthyroid mice that received short TH treatment at adult stage as in the present study (28). There were, however, few similarities in the TH response of glial cells (Supplementary Table S3) (24). Furthermore, when we compare this gene list to the lists present in a database gathering all recent transcriptome analysis of TH response in various tissues and cell types (26), only a few similarities were observed. Overall, we found very limited overlap between our and these datasets (97/653 genes). Therefore, most of the T3-responsive genes appear to be novel and different from those reported in other situations or cell types.

### Bulk RNA-seq of Sorted Cells: Hypothalamus Astrocytes

**Figure 4.**
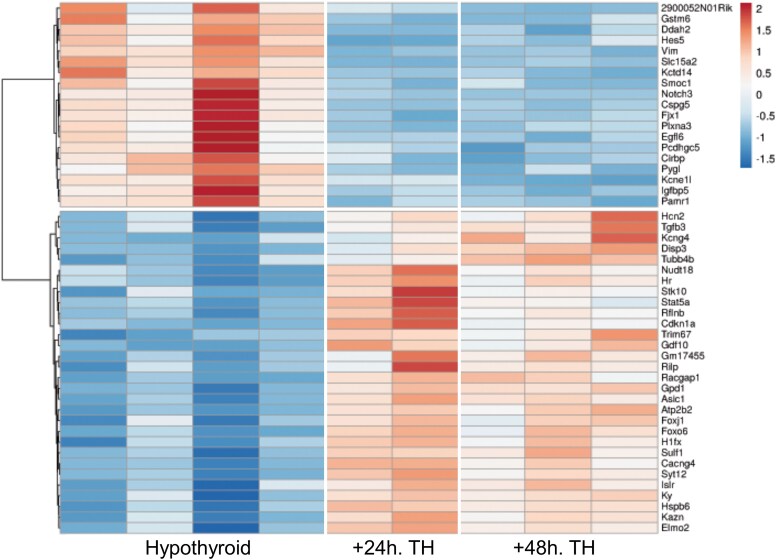
Bulk RNA-seq analysis of hypothalamic astrocyte response to TH stimulation. The heatmap (red for high expression, blue for low expression) presents the 50 genes with the highest fold-change among 850 DEG detected after the RNA-seq analysis of sorted hypothalamic astrocytes. Note that the expression of DEGs does not change significantly between 24 hours and 48 hours of TH treatment.

The main drawback of snRNA-seq is the low sequencing depth/nucleus, which limits the sensitivity of the analysis in a given cell type and the capacity to identify differential expression. To overcome this limitation, we treated hypothyroid mice with TH, as above, and performed bulk RNA-seq from sorted astrocytes and GABAergic neurons. In each case, we identified the expression of more than 60% of the annotated genes, while this ratio was only 9% in our snRNA-seq, confirming that bulk analysis of sorted cells provides a much deeper analysis of the transcriptome. For astrocytes, we used magnetic sorting to isolate Glast + cells from the hypothalamus and analyzed whole cell RNA. Enrichment was evaluated *a posteriori* by ranking the genes according to their expression level and comparing with a previously generated whole hypothalamus RNA-seq (not shown). This highlighted a strong enrichment for *Slc1a2/Glast* and *Gfap* astrocytes markers and a depletion of neuronal and oligodendroglial markers (*Gad1, Gad2, Mag*). No obvious enrichment was observed for tanycytes (*Rax, Col23a1*) and microglia *(Runx, Dock8)* which also express the *Slc1a2/Glast* gene. In summary, this bulk RNA-seq essentially reflects TH regulation of gene expression in astrocytes. We also extended the TH treatment to 48 hours for some mice, expecting a more robust response. Although the protocols differ at several levels, bulk RNA-seq analysis confirmed most of the previous observations in astrocytes and significantly extended the list of DEGs (Supplementary Table S4) (24). Extending TH treatment to 48 hours did not lead to a stronger response (Fig. 4).

### Bulk RNA-seq of Sorted Nuclei: Hypothalamus GABAergic Neuron Nuclei

In order to isolate nuclear RNA from the GABAergic neurons, we used transgenic mice expressing a nuclear fluorescent protein and ran nuclei suspensions prepared from hypothalamus in a Fluorescence-Activated Cell Sorter before RNA extraction. By contrast, this bulk RNA-seq identified a very small number of DEGs (Supplementary Table S4) (24). Few changes in gene expression were visible at whole hypothalamus level by RT-qPCR. Interestingly, these included the downregulation of the *Htr3b* gene, which is only expressed in a very small number of GABAergic neurons within Cluster 1 and was only identified as DEG by bulk RNA-seq (Supplementary Fig. S3) (24).

## Discussion

Combining single nuclei and bulk RNA-seq analysis, we list genes whose expression is up- or downregulated when TH levels are restored in hypothyroid mice. The present atlas (Supplementary Table S1) aims to support future research on the very complex influence of T3 on hypothalamic functions. One main conclusion is that the repertoire of genes varies widely across cell types, even within subcategories of neurons. Our unsuccessful attempt to find overlaps between the TH response of cortical and hypothalamic cell types further reinforces the view that different cell types display a very different response to TH stimulation. This opens a broad avenue for later investigations that should help to understand both the molecular basis of the diverse cellular responses and their physiological consequences.

Although this is better documented in other areas of the brain, T3 has an important function during the development of the hypothalamus (29). This early influence of T3 is probably the main explanation for the divergence between our data, in which neurodevelopment is not affected, and the data previously obtained from mice in which the dominant-negative TRα1^R384C^ receptor was present since embryonic life (27). The main defect of the hypothalamus of these mice is a defect in oligodendrocytes differentiation, which is consistent with that well-documented observation that differentiation of oligodendrocyte progenitors, which takes several days, is dependent on TH (30, 31). On the contrary, according to our results, differentiated oligodendrocytes display limited sensitivity to TH stimulation after terminal maturation and express the *Thra* and *Thrb* genes at a low level. However, the action of TH might influence the function of these cells. In particular, the TH-responsive gene *Hcn2*, which encodes the cyclic nucleotide-gated 2 ion channel, is known to influence myelin sheath length and conduction along myelinated axons (32).

Beyond this example, it is difficult to infer from our results the many consequences that the unveiled TH regulations might have on hypothalamic neuronal networks. This is most notably because the nuclear fraction of RNA that we analyzed contains many noncoding RNA and unannotated transcripts of unknown functions. For example, one of the main downregulated gene is BC1, which produces a noncoding RNA with elusive function (33, 34). Attempts to use gene ontology analysis to highlight pathways and cellular functions in which TH could exert a coordinated influence were for the most part unsuccessful. The exception was astrocytes, for which both single nuclei and bulk RNA-seq highlighted a peculiar sensitivity to TH stimulation. In these cells, we identified a large set of genes upregulated by TH that encode calcium or potassium transporters (GO:0034765; GO:0015079). In particular, bulk RNA-seq identified 15 genes encoding potassium channels (GO:0015079) out of 336 upregulated genes, which represents a 6.5-fold enrichment (*P* value: 6 × 10^−9^) over the expected ratio. This raises the interesting hypothesis that T3 regulates the capacity of astrocytes to rectify potassium concentration near synapses and plays a role in maintaining normal neuronal excitability. This upregulation of genes encoding potassium channels is concomitant with a negative response of genes encoding extracellular matrix components (GO:0062023; enrichment 3.2-fold; *P* value 10^−6^). This implies that TH might also regulate the capacity of astrocytes to participate in the breakdown, synthesis, and remodeling of the extracellular matrix around neurons (35). It has recently been shown that a specific type of extracellular matrix, called the perineuronal net, plays a major role in stabilizing the synapses of some hypothalamic neurons and can modulate the activity of the neuronal network (36). Thus, TH might interfere with these regulations by acting in astrocytes. These are only a few speculations suggested by these new data, and we expect that this atlas will help other investigators to elaborate additional hypotheses on the mechanism of TH action in the hypothalamus.

### Limitations

The ultimate aim of this project is to unravel the molecular mechanisms by which TH regulate the functions of the hypothalamus. A prerequisite is to identify the TR target genes in all types of hypothalamic cells, that is, the genes which transcription is directly regulated by the T3-bound TRα1 and TRβ1/2 receptors. As we found an equivalent number of genes upregulated or downregulated by TH, while TRs are mainly transcription activators, many of the TH-responsive genes that we describe might not be under direct transcriptional control of TRs. An important step would be to address chromatin occupancy by TRs on a genome-wide scale and single-cell level, which is not currently possible. Our understanding of the hypothalamic response to TH would also benefit from a deeper analysis of the single nuclei. Scaling up the analysis to a much larger number of cells would increase its statistical power and identify additional differentially expressed genes in rare cell types. A time course analysis might also establish a hierarchy between early and late response, and help to better pinpoint the initial events stimulated by TH. Notably, our single nuclei analysis did not capture the negative regulation of the *Trh* gene expression by TH in the hypophysiotrophic neurons of the paraventricular nucleus (37), which reaches a maximum after several days of TH treatment (38). Finally, as we pooled samples from males and females, our data cannot be used to analyze the interaction between sex and TH response.

## Data Availability

The raw data are available online In BioStudies (ArrayExpress), referenced E-MTAB-14810 for single nuclei RNAseq (fastq and processed files resulting of the Seurat V5 analyses), E-MTAB-15049 for bulk RNA-seq from hypothalamus astrocytes, and E-MTAB-15050 for bulk RNA-seq from hypothalamus GABAergic neurons. The scripts used to perform the snRNA-seq analyses are available at https://gitbio.ens-lyon.fr/igfl/psi/snRNAseq_hypothyro_short_reads. Supplementary figures and tables are available at *figshare:*  https://doi.org/10.6084/m9.figshare.28804142.v1.

## Abbreviations

DEG: differentially expressed gene
GFP: green fluorescent protein
NSB: Nuclei Suspension Buffer
RT-qPCR: quantitative reverse transcriptase polymerase chain reaction
snRNA: single nuclei RNA
T3: triiodothyronine
T4: thyroxine
TH: thyroid hormone (T3 and T4)
TR: thyroid hormone nuclear receptor
TRH: thyrotropin-releasing hormone
UMAP: uniform manifold approximations and projections

## References

[1] Pearson CA, Placzek M. Development of the medial hypothalamus: forming a functional hypothalamic-neurohypophyseal interface. Curr Top Dev Biol. 2013;106:49‐88. 24290347 10.1016/B978-0-12-416021-7.00002-X

[2] Ortiga-Carvalho TM, Chiamolera MI, Pazos-Moura CC, Wondisford FE. Hypothalamus-pituitary-thyroid axis. Compr Physiol. 2016;6(3):1387‐1428. 27347897 10.1002/cphy.c150027

[3] Lopez M, Alvarez CV, Nogueiras R, Dieguez C. Energy balance regulation by thyroid hormones at central level. Trends Mol Med. 2013;19(7):418‐427. 23707189 10.1016/j.molmed.2013.04.004

[4] Sentis SC, Dore R, Oelkrug R, Kolms B, Iwen KA, Mittag J. Hypothalamic thyroid hormone receptor alpha1 signaling controls body temperature. Thyroid. 2024;34(2):243‐251. 38149585 10.1089/thy.2023.0513

[5] Wu Z, Martinez ME, DeMambro V, Francois M, Hernandez A. Developmental thyroid hormone action on pro-opiomelanocortin-expressing cells programs hypothalamic BMPR1A depletion and brown fat activation. J Mol Cell Biol. 2023;14(9):mjac078. 36581316 10.1093/jmcb/mjac078 PMC9982511

[6] Coppola A, Liu ZW, Andrews ZB, et al A central thermogenic-like mechanism in feeding regulation: an interplay between arcuate nucleus T3 and UCP2. Cell Metab. 2007;5(1):21‐33. 17189204 10.1016/j.cmet.2006.12.002 PMC1783766

[7] Dardente H, Hazlerigg DG, Ebling FJ. Thyroid hormone and seasonal rhythmicity. Front Endocrinol (Lausanne). 2014;5:19. 24616714 10.3389/fendo.2014.00019 PMC3935485

[8] de Vries EM, Nagel S, Haenold R, et al The role of hypothalamic NF-kappaB signaling in the response of the HPT-axis to acute inflammation in female mice. Endocrinology. 2016;157(7):2947‐2956. 27187176 10.1210/en.2016-1027

[9] Sinko R, Mohacsik P, Kovari D, et al Different hypothalamic mechanisms control decreased circulating thyroid hormone levels in infection and fasting-induced non-thyroidal illness syndrome in male thyroid hormone action indicator mice. Thyroid. 2023;33(1):109‐118. 36322711 10.1089/thy.2022.0404 PMC9885537

[10] Hernandez A, Quignodon L, Martinez ME, Flamant F, St Germain DL. Type 3 deiodinase deficiency causes spatial and temporal alterations in brain T3 signaling that are dissociated from serum thyroid hormone levels. Endocrinology. 2010;151(11):5550‐5558. 20719855 10.1210/en.2010-0450 PMC2954712

[11] Yen PM, Ando S, Feng X, Liu Y, Maruvada P, Xia X. Thyroid hormone action at the cellular, genomic and target gene levels. Mol Cell Endocrinol. 2006;246(1–2):121‐127. 16442701 10.1016/j.mce.2005.11.030

[12] Richard S, Guyot R, Rey-Millet M, et al A pivotal genetic program controlled by thyroid hormone during the maturation of GABAergic neurons. iScience. 2020;23(3):100899. 32092701 10.1016/j.isci.2020.100899 PMC7037980

[13] Kriaucionis S, Heintz N. The nuclear DNA base 5-hydroxymethylcytosine is present in purkinje neurons and the brain. Science. 2009;324(5929):929‐930. 19372393 10.1126/science.1169786 PMC3263819

[14] Galaxy C . The Galaxy platform for accessible, reproducible, and collaborative data analyses: 2024 update. Nucleic Acids Res. 2024;52(W1):W83‐W94. 38769056 10.1093/nar/gkae410 PMC11223835

[15] Langmead B, Trapnell C, Pop M, Salzberg SL. Ultrafast and memory-efficient alignment of short DNA sequences to the human genome. Genome Biol. 2009;10(3):R25. 19261174 10.1186/gb-2009-10-3-r25 PMC2690996

[16] Anders S, Pyl PT, Huber W. HTSeq--a Python framework to work with high-throughput sequencing data. Bioinformatics. 2015;31(2):166‐169. 25260700 10.1093/bioinformatics/btu638 PMC4287950

[17] Love MI, Huber W, Anders S. Moderated estimation of fold change and dispersion for RNA-seq data with DESeq2. Genome Biol. 2014;15(12):550. 25516281 10.1186/s13059-014-0550-8 PMC4302049

[18] Metsalu T, Vilo J. ClustVis: a web tool for visualizing clustering of multivariate data using Principal Component Analysis and heatmap. Nucleic Acids Res. 2015;43(W1):W566‐W570. 25969447 10.1093/nar/gkv468 PMC4489295

[19] Hao Y, Stuart T, Kowalski MH, et al Dictionary learning for integrative, multimodal and scalable single-cell analysis. Nat Biotechnol. 2024;42(2):293‐304. 37231261 10.1038/s41587-023-01767-y PMC10928517

[20] McGinnis CS, Murrow LM, Gartner ZJ. DoubletFinder: doublet detection in single-cell RNA sequencing data using artificial nearest neighbors. Cell Syst. 2019;8(4):329‐337 e324. 30954475 10.1016/j.cels.2019.03.003 PMC6853612

[21] Korsunsky I, Millard N, Fan J, et al Fast, sensitive and accurate integration of single-cell data with harmony. Nat Methods. 2019;16(12):1289‐1296. 31740819 10.1038/s41592-019-0619-0 PMC6884693

[22] Steuernagel L, Lam BYH, Klemm P, et al HypoMap-a unified single-cell gene expression atlas of the murine hypothalamus. Nat Metab. 2022;4(10):1402‐1419. 36266547 10.1038/s42255-022-00657-y PMC9584816

[23] Bookout AL, Cummins CL, Mangelsdorf DJ, Pesola JM, Kramer MF. High-throughput real-time quantitative reverse transcription PCR. Curr Protoc Mol Biol. 2006;Chapter 15:Unit 15 18. 10.1002/0471142727.mb1508s73 18265376

[24] Wu S . Suppl material “An atlas of thyroid hormone responsive genes in adult mouse hypothalamus”. 10.6084/m9.figshare.28804142.v1. PMC12068221 40302247

[25] Yao Z, van Velthoven CTJ, Kunst M, et al A high-resolution transcriptomic and spatial atlas of cell types in the whole mouse brain. Nature. 2023;624(7991):317‐332. 38092916 10.1038/s41586-023-06812-z PMC10719114

[26] Zekri Y, Guyot R, Flamant F. An atlas of thyroid hormone receptors’ target genes in mouse tissues. Int J Mol Sci. 2022;23(19):11444. 36232747 10.3390/ijms231911444 PMC9570117

[27] Sreenivasan VKA, Dore R, Resch J, et al Single-cell RNA-based phenotyping reveals a pivotal role of thyroid hormone receptor alpha for hypothalamic development. Development. 2023;150(3):dev201228. 36715020 10.1242/dev.201228 PMC10110490

[28] Hochbaum DR, Hulshof L, Urke A, et al Thyroid hormone remodels cortex to coordinate body-wide metabolism and exploration. Cell. 2024;187(20):5679‐5697.e5623. 39178853 10.1016/j.cell.2024.07.041 PMC11455614

[29] Alkemade A . Thyroid hormone and the developing hypothalamus. Front Neuroanat. 2015;9:15. 25750617 10.3389/fnana.2015.00015 PMC4335174

[30] Billon N, Jolicoeur C, Tokumoto Y, Vennstrom B, Raff M. Normal timing of oligodendrocyte development depends on thyroid hormone receptor alpha 1 (TRalpha1). Embo J. 2002;21(23):6452‐6460. 12456652 10.1093/emboj/cdf662 PMC136965

[31] Picou F, Fauquier T, Chatonnet F, Flamant F. A bimodal influence of thyroid hormone on cerebellum oligodendrocyte differentiation. Mol Endocrinol. 2012;26(4):608‐618. 22361821 10.1210/me.2011-1316 PMC5417134

[32] Swire M, Assinck P, McNaughton PA, Lyons DA, Ffrench-Constant C, Livesey MR. Oligodendrocyte HCN2 channels regulate myelin sheath length. J Neurosci. 2021;41(38):7954‐7964. 34341156 10.1523/JNEUROSCI.2463-20.2021 PMC8460148

[33] Lewejohann L, Skryabin BV, Sachser N, et al Role of a neuronal small non-messenger RNA: behavioural alterations in BC1 RNA-deleted mice. Behav Brain Res. 2004;154(1):273‐289. 15302134 10.1016/j.bbr.2004.02.015

[34] Skryabin BV, Sukonina V, Jordan U, et al Neuronal untranslated BC1 RNA: targeted gene elimination in mice. Mol Cell Biol. 2003;23(18):6435‐6441. 12944471 10.1128/MCB.23.18.6435-6441.2003 PMC193692

[35] Zhang N, Song B, Bai P, et al Perineuronal nets’ role in metabolism. Am J Physiol Endocrinol Metab. 2024;327(4):E411‐E421. 39140971 10.1152/ajpendo.00154.2024

[36] Beddows CA, Shi F, Horton AL, et al Pathogenic hypothalamic extracellular matrix promotes metabolic disease. Nature. 2024;633(8031):914‐922. 39294371 10.1038/s41586-024-07922-y PMC11424483

[37] Fekete C, Lechan RM. Central regulation of hypothalamic-pituitary-thyroid axis under physiological and pathophysiological conditions. Endocr Rev. 2014;35(2):159‐194. 24423980 10.1210/er.2013-1087 PMC3963261

[38] Wondisford FE . Evaluating the hypothalamic-pituitary-thyroid (HPT) axis in mice. Methods Mol Biol. 2018;1801:155‐161. 29892823 10.1007/978-1-4939-7902-8_13

